# Cryptotanshinone Suppressed Postmenopausal Osteoporosis by Preventing RANKL-Mediated Osteoclastogenesis against Kidney Injury

**DOI:** 10.1155/2022/2821984

**Published:** 2022-01-29

**Authors:** Wenjiu Yang, Jing Han, Shuo Gong, Jun Zhao, Tengbo Yu, Jinfeng Ma

**Affiliations:** ^1^Department of Spinal Surgery, The Affiliated Hospital of Qingdao University, Qingdao 266003, Shandong, China; ^2^Department of Ophthalmology, The Affiliated Hospital of Qingdao University, Qingdao 266003, Shandong, China; ^3^Department of Spine Surgery, First Affiliated Hospital of Shandong First Medical University (Shandong Province Qianfoshan Hospital), Jinan, Shandong, China; ^4^Department of Pharmacy, The Affiliated Hospital of Qingdao University, Qingdao, Shandong, China; ^5^Department of Sports Medicine, The Affiliated Hospital of Qingdao University, Qingdao, Shandong, China

## Abstract

**Background:**

Cryptotanshinone (CPT), an active component extracted from the root of *Salvia miltiorrhiza* Bunge, exhibits extensive favorable bioactive properties including anti-inflammatory, antioxidative, antibacterial, and antitumor effects. This study aims to investigate the effects of CPT on osteogenesis and explore related mechanisms both in vivo and in vitro.

**Methods:**

In the in vivo experiment, ovariectomized (OVX) female rats were intragastrically administered with CPT at doses of 10 mg/kg and 20 mg/kg for 13 consecutive weeks. Dual-energy X-ray absorptiometry was employed to detect bone mineral density (BMD). ELISA assay was leveraged to detect the biochemical parameters such as BUN and creatinine in the kidney samples. Bone and kidney sections were dyed by H&E and Masson staining kits. In the in vitro experiment, the RAW 264.7 cells were stimulated through the receptor activation of the nuclear factor kappa B ligand (RANKL) to establish an osteoclast differentiation model, and CPT's protective effect against bone loss was evaluated. Differentiated osteoclasts were determined by TRAP staining. While, osteoclast-marker proteins such as NFATc1, c-Fos, and cathepsin K were identified by Western blot.

**Results:**

The results from in vivo experiments revealed that CPT could elevate bone mass and increase bone formation markers in OVX rats. Intriguingly, CPT administration noticeably ameliorated the kidney injury in OVX rats by suppressing BUN and restoring creatinine levels. Furthermore, the results from in vitro experiments suggested that CPT downregulated the protein expression of osteoclast-associated genes such as cathepsin K, c-Fos, and NFATc1 which hinted the related potential mechanisms.

**Conclusion:**

The evidence from in vivo and in vitro experiments suggested that CPT exerted antiosteoclastogenic effects by inhibiting the activation of osteoclast differentiation followed by suppressing the protein expressions of cathepsin K, c-Fos, and NFATc1 in osteoclast precursors, and it exhibited protective effects against kidney damage, which highlighted its advantage in clinical application.

## 1. Background

Nowadays, millions of people suffer from osteoporosis, a metabolic bone disease accompanied by the decrease of bone mineral content and bone imbalance [1, 2]. The mechanisms underlying osteoporosis are sophisticated, involving aging, calcium malabsorption, endocrine disorders, and limb degeneration, as well as immune and genetic factors [1, 3]. Postmenopausal osteoporosis is one of the most common bone diseases [4]. In postmenopausal women, osteoporotic fractures are more common than other diseases such as stroke, myocardial infraction, and breast cancer [5, 6]. Females account for more than 70% of the total patients suffering from this disease due to the hormone level decline associated with aging [7, 8]. As estrogen regulates factors associated with the balance of bone formation and absorption [6], postmenopausal women are at high risk of developing osteoporosis owing to estrogen deficiency. Estrogen replacement therapy, the most common therapy for the prevention and treatment of postmenopausal osteoporosis [9, 10], has long been evidenced to be correlated with elevated risks of breast cancer, ovarian cancer, endometrial cancer, and cardiovascular diseases in postmenopausal women [11, 12]. Therefore, estrogen therapy is not the optimal option to prevent fractures in postmenopausal women, and it is imperative to creatively develop effective and efficient treatment strategies in this regard.

It is well-known that natural products and derivatives have inherent biochemical, biomechanical, and structural functionalities and harbor excellent biocompatibility and biodegradability, which make them promising candidates in terms of preventing the development of metabolic disorders and diseases [13, 14]. Cryptotanshinone (CPT), an active component extracted from the root of *Salvia miltiorrhiza* Bunge, exhibits substantial favorable properties including anti-inflammatory, antioxidative, antibacterial, and antitumor effects [15, 16]. Remarkably, CPT is proven to be able to relieve the cartilage destruction and recover the thickening of the subchondral bone in osteoarthritis mouse models, which demonstrates its potential treatment effect against bone disorder [17, 18]. Nevertheless, its underlying molecular mechanism in osteoclastogenesis remains unexplored. In the present study, we aimed to explore the effects of CPT on osteoporosis and clarify the possible mechanism in mediating osteoclast differentiation.

In this research, we designed in vivo and in vitro experiments to identify the treatment effect of CPT on osteoporosis and its related mechanisms. Our team revealed that CPT exerted an ameliorative effect on the progress of osteoporosis via mediating the metabolism of osteoclast, exhibiting protective effects on the kidneys. These data provided a theoretical foundation to unravel the potential molecular mechanisms of CPT against osteoporosis.

## 2. Materials and Methods

### 2.1. Materials

CPT was purchased from Dongguan Hanshuo Jianyuan Biotechnology (Shenzhen, China), while RANKL was purchased from R&D Systems (Minneapolis, MN, USA) and dissolved in phosphate-buffered saline. The ELISA kits, the detection of blood urea nitrogen (BUN), creatinine, ALP, OPG, and RANKL were purchased from Wuhan Huamei Biological Co., Ltd. (Wuhan, China). Anti-NFATc1, anticathepsin K and anti-c-Fos antibodies were purchased from Cell Signaling Technology (the USA), and H&E and Masson dying kits were purchased from Nanjing Jiancheng Biological Engineering Research Institute (Nanjing, China). JYB1-1 calcium removal solution and protein extraction kit were purchased from Solarbio Company (Beijing, China). The tartrate-resistant acid phosphatase (TRAP) staining kit was obtained from Whatman (England), and the MTT assay kit was purchased from Abcam (England).

### 2.2. Animals

All animal experiments were conformed to the NIH guideline for the Care and Use of Laboratory Animals (NIH Publications No. 80–23, revised 1978). All animal experiments were carried out in a strict accordance with the regulations for the Care and Use of Laboratory Animals of the National Institute of Animal Health and the guidance by the Ethics Committee of Qingdao University (animal welfare assurance number:14–0027). Healthy specific pathogen free (SPF) female Sprague Dawley rats (3 months old) were provided by Spaifu Biotechnology Co. Ltd. (Beijing, China; license number: SCXK(Beijing) 2016–0002). All rats were raised in polypropylene cages with sterile paddy husks and kept under a controlled environment (humidity 50–60%; ambient temperature 24 ± 1 °C; light-dark cycle: 12L : 12D). The rats were randomly assigned into the following groups: control (*n* = 8, sham operation), osteoporosis (*n* = 8), osteoporosis plus low-dose CPT (10 mg/kg, *n* = 8), and osteoporosis plus high-dose CPT (20 mg/kg, *n* = 8). After the 4-week feeding period, all rats were fasted overnight and anaesthetized with diethyl ether. The femur samples were removed by gauze and fixed in 4% paraformaldehyde overnight at 4°C and then stored in 70% ethanol for micro-CT scanning. The trabecular region of interest was selected and analyzed via quantifying bone mineral density (BMD), bone mineral content (BMC), and morphometric calculations. The following direct trabecular metric parameters were measured: bone volumetric fraction (BV/TV%), trabecular thickness (Tb Th, mm), trabecular separation (Tb Sp, mm), and trabecular number (Tb N, 1/mm). The kidneys were excised and stained with H&E. Subsequently, the bone sections of each group were dyed by H&E and Masson as per the supplier's instructions.

### 2.3. ELISA Assay

The secretion levels of urea nitrogen (BUN) and creatinine in sera were determined using ELISA kits following the instructions of the manufacturer. The assay of each specimen was performed in triplicate, the average of which was identified.

### 2.4. Network Pharmacology Predicted the Mechanism of CPT in Treating Osteoporosis

Inputting CPT into the symptom and target-screening SymMap (http://www.symmap.org/) database, we obtained 498 symptoms and 59 targets. In this network, we selected the symptoms of osteoporosis and targets involved in osteoclast differentiation.

### 2.5. Cell Culture

The RAW 264.7 cell line was obtained from American Type Culture Collection (ATCC, Rockville, the USA). Cells were cultured in DMEM supplemented with 10% FBS, penicillin (100 *µ*g/mL), and streptomycin (100 *µ*g/mL) in a humidified incubator with 5% CO_2_ at 37°C.

### 2.6. MTT Assay

RAW 264.7 cells were cultured on confocal dishes at a density of 1×104 cells/mL overnight and then added with CPT (0.1, 1, 10, and 100 *µ*M) for one hour. Subsequently, we detected the cell viability following the kit instruction.

### 2.7. TRAP Staining

Differentiated osteoclasts were determined by TRAP staining following the manufacturer's protocol. On the 5th day, the coverslip was removed from the plate, and cells were fixed with 4% paraformaldehyde in PBS for 20 minutes at room temperature and then washed with PBS. After natural drying, the TRAP fixation solution was fixed for 3 minutes at 4°C and slightly dried after washing. TRAP incubation solution was added and placed in an incubator without light at 37°C for 60 minutes. Afterwards, plants were dyed with hematoxylin solution for 3–5 minutes and later washed and dried for microscopic examination (Olympus Corporation of the Americas, Waltham, MA, ix81). Finally, TRAP-positive multinucleated cells (purple) containing at least 5 nuclei were counted as osteoclasts.

### 2.8. Western Blotting

RAW 264.7 cells were cultured on confocal dishes at a density of 1×104 cells/mL overnight and then added with CPT (1, 10 *µ*M) for one hour. For RAW 264.7 cells, cells were seeded into culture dishes and incubated overnight. After treatment, these cells were collected and proteins were extracted, with protein concentrations quantified using BCA protein assay kits. The samples were denatured at 95°C for 10 min and then isolated with 8% SDS-PAGE. Subsequently, proteins were transferred onto PVDF membranes and blocked with 5% nonfat milk followed by the incubation with primary antibodies GAPDH (1 : 1000), NFATc1(1 : 500), cathepsin K (1 : 500), and c-Fos (1 : 500) overnight at 4°C. After the incubation with secondary antibodies for 2 h at room temperature, chemiluminescence signals were detected with the ChemiDoc^TM^ Imager.

### 2.9. Statistical Analyses

We presented all data by the averages and standard deviations of the averages (SD). Differences within groups were analyzed statistically using one-way ANOVA, with *p* < 0.05 considered to be statistically significant. All analyses were performed using SPSS 17.0 (Chicago, IL, the USA) and GraphPad Prism 6.0 (GraphPad Software, Inc., La Jolla, CA, the USA).

## 3. Results

### 3.1. Effect of CPT on OVX-Induced Osteoporosis in Rats

**Figure 1:**
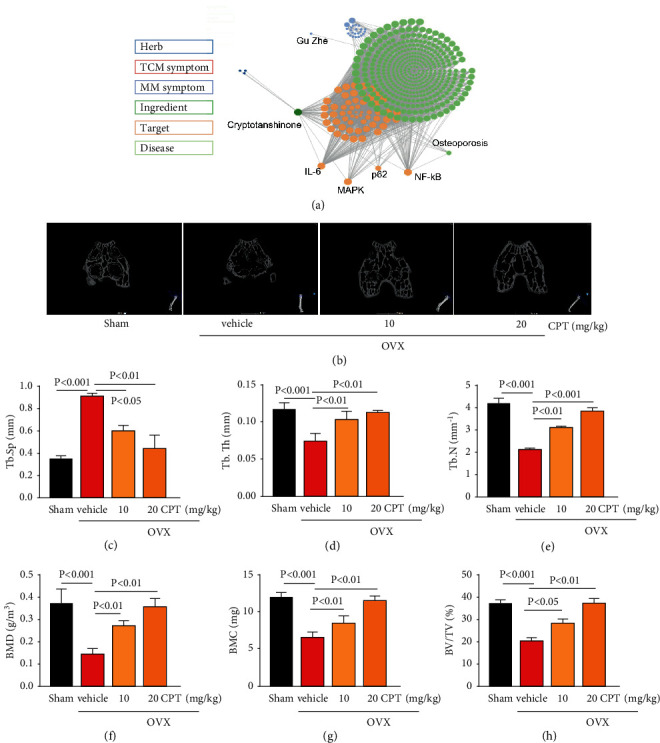
Effect of CPT on OVX-induced bone microarchitecture. (a) Data from network pharmacology analysis. The rats were randomly divided into four groups (*n* = 10) and treated as described in the section of Materials and Methods: Sham group, OVX group, and OVX groups intragastrically receiving CPT at 10 and 20 mg/kg/day, respectively. (b) Representative micro-CT images. Femur surfaces were color coded to indicate the local thickness (0–0.800 mm). Characteristics of bone microarchitecture in femur including (c) trabecular separation (Tb.Sp,mm), (d) trabecular thickness (Tb.Th, mm), (e) trabecular number (Tb.N, mm−1), (f) bone mineral density (BMD, g/m3), (g) bone mineral content (BMC, mg), and (h) bone volume fraction(BV/TV%) were detected.

**Figure 2:**
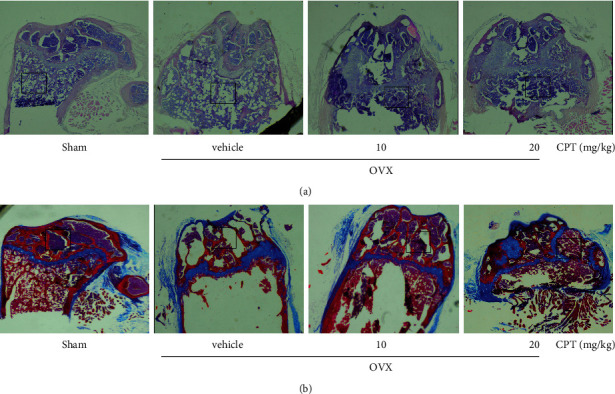
Effect of CPT on OVX-induced bone loss. (a) Morphological changes of bone loss in femur per group detected by H&E staining(magnification ×40). (b) Masson staining for femur (magnification ×40). Remarkable changes are marked in the black box.

On the foundation of our network pharmacology-based prediction, CPT exhibited a potential in reversing bone metabolism disorders (Figure 1(a)). To identify the prediction from network pharmacologic analysis that CPT displayed a potential in improving osteoporosis, the effects of CPT on osteoporosis were evaluated in vivo. First, the rat model of OVX-induced osteoporosis was established. Second, the data analysis from micro-CT revealed a significant decrease in bone mineral density (BMD), bone mineral content (BMC), bone volume fraction (BV/TV), trabecular separation (Tb Sp), trabecular thickness (Tb Th), and trabecular number (Tb N), whereas an increase in trabecular separation (Tb Sp) was identified. These variations are important characteristics of microarchitectural deterioration of tissue, which were significantly suppressed by CPT treatment (Figures 2(b)–2(h)). Taken together, the data herein indicated the potential of CPT in ameliorating osteoporosis.

### 3.2. CPT Protected the Morphological Deterioration in OVX Rats

Morphologically, bone loss and degeneration were remarkably observed in the femur of the OVX group, while significant inhibition of bone mass loss was observed in the rats treated with CPT by H&E staining (Figure 2(a)). The maturity of bone tissue was evaluated via Masson staining. Normally, the type 1 and 2 collagen is balanced in healthy bone tissue, while the osteoporotic tissue is deficient in new collagen due to the disorder of the bone metabolism. The result shows that CPT administration reversed the reduction of collagen formation (Figure 2(b)). Collectively, the above results reveal that CPT could improve bone homeostasis in OVX rats.

### 3.3. CPT Reversed OVX-Induced Kidney Damage Secondary to Osteoporosis

**Figure 3:**
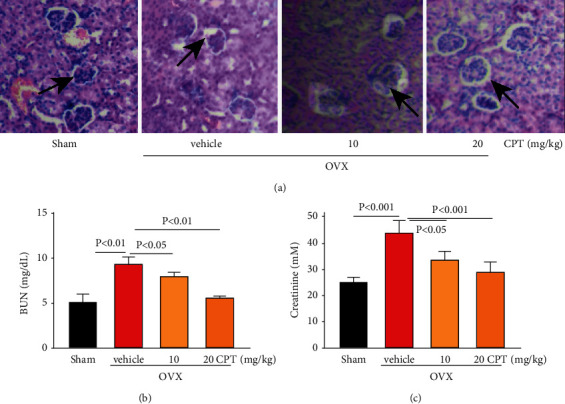
Effect of CPT on OVX-induced kidney failure. The kidneys were isolated from rats of the Sham, OVX, and OVX rats with CPT (10 and 20 mg/kg/day) groups. (a) Morphological change in glomerulus via H&E staining. The releasing of (b) BUN and (c) creatinine detected by ELISA kits. The black arrow points to atrophic glomeruli.

Kidney failure is one of the most severe side effects arising from current clinical agents [19]. Intriguingly, CPT treatment remarkably suppressed chronic kidney injury by ameliorating glomerular atrophy (Figure 3(a)), decreasing BUN release level, and restoring creatinine level in OVX rats (Figures 3(b) and 3(c)), which demonstrated its advantage in clinical application.

### 3.4. CPT Exhibited No Toxicity on Macrophages and Suppressed Osteoclast Differentiation

**Figure 4:**
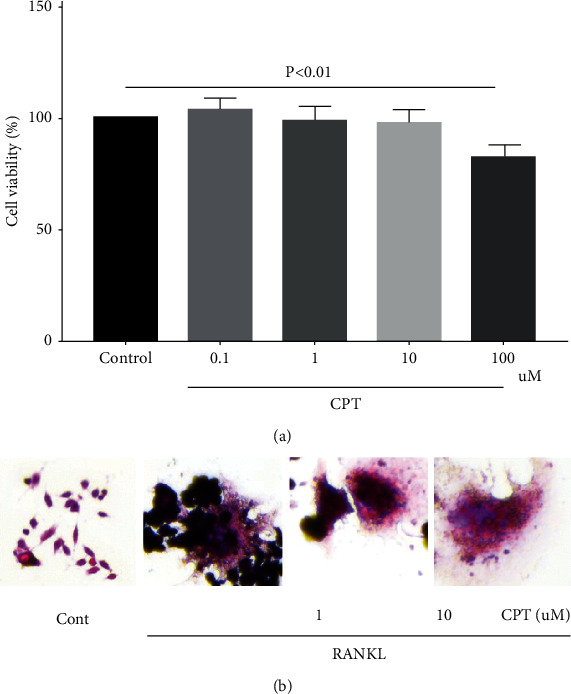
(a) CPT exhibiting no toxicity to RAW 264.7 and reversed differentiation induced by RANKL. First, RAW 264.7 cells were cultured for toxicology trial with different concentrations of CPT (0, 0.1, 1, 10, and 100 *µ*M). (b) Multinucleated cells with more than five nuclei, which were considered mature osteoclasts, observed under a light microscope after RANKL treatment, and they were suppressed by CPT cotreatment.

As shown in Figure 4(a), CPT almost exhibited no toxicity at doses below 10 *µ*M, which proved that its safety characteristic substances in bone tissues and osteocytes were determined by the staining of TRAP, a marker enzyme of osteoclasts, via making osteoclasts red. Evidently, CPT suppressed TRAP-positive osteoclasts in the process of osteoclast differentiation. Collectively, CPT inhibited osteoclast differentiation (Figure 4(b)).

### 3.5. CPT Suppressed RANKL-Induced Osteoclast-Specific Protein Expression

**Figure 5:**
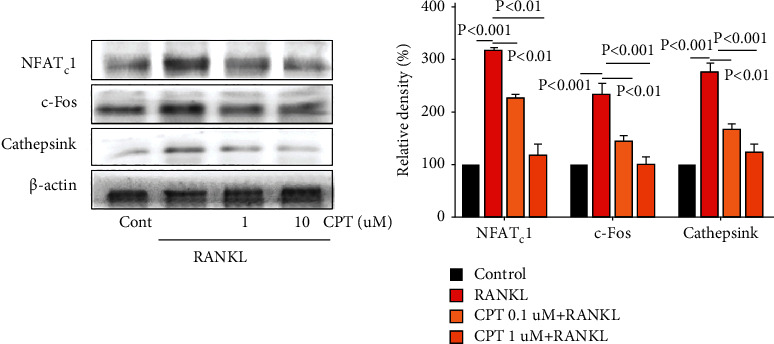
Role of osteoclast differentiation pathway in the process of CPT treatment-attenuated osteoclast differentiation. RAW 264.7 cells were pretreated with CPT (1, 10 *μ*M) for 1 h in response to RANKL (50 ng/mL) for 5 days, and the expression of NFATc1, c-Fos, and cathepsin K was detected by Western blotting.

RANKL is crucial for the differentiation and activation of osteoclast [2]. RANKL-induced osteoclastogenesis is associated with changes in the expression of osteoclast-specific genes. In the in vitro study, the effect of CPT on osteoclastogenesis was detected by evaluating the expression of osteoclast specific protein such as cathepsin K, NFATc1, and c-Fos by Western blotting. CPT effectively suppressed the high expression level induced by RANKL, which hinted that CPT inhibited osteoclastogenesis by repressing the osteoclast-specific protein expression (Figure 5).

## 4. Discussion

In recent years, osteoporosis has become a serious healthcare concern for the aging population [20, 21]. It is projected that more than about 50% of all osteoporotic hip fractures will occur in Asia by the year 2050. Specially in populous countries like China and India, the majority of the population living in rural areas have less access to diagnostics and treatment compared to urban areas. This suggests that the number of people with osteoporosis may be underestimated in rural areas throughout the Asian countries [22, 23]. The strength and integrity of the bones are tightly regulated by bone-forming osteoblasts and bone-resorpting osteoclasts [19, 24]. Increased bone resorption by osteoclasts is a manifestation of several lytic bone diseases [25]. Natural plant compounds have been reported to be able to improve bone health, stimulate bone formation, and block pathological bone loss [19, 26].

CPT, extracted from *Salvia miltiorrhiza* Bunge [27, 28], which is a well-known natural traditional Chinese medicine [29, 30], has been reported that it can prevent retinoic acid-induced bone loss in rats [31, 32]. Estrogen deficiency and inflammation are known to play vital roles in the bone metabolism and occurrence of osteoporosis [33, 34]. Remarkably, inflammation plays an essential role in disease development via the mediating metabolism [35, 36]. At present, the effects of CPT on estrogen deficiency-induced bone loss and related mechanisms are poorly understood. In this research, we selected OVX rats to establish a model of postmenopause osteoporosis (PMOP). First, our team proved that CPT treatment could reverse the transnormal femoral BMD and biomarkers of bone formation and resorption of OVX rats. These results implied implying that CPT could be remarkably beneficial to patients with PMOP and exhibited an evident protective effect against kidney damage secondary to osteoporosis.

Next, in vitro studies revealed the mechanisms of CPT in improving osteoporosis. Osteoclasts are key mediators of skeletal diseases [37, 38], the differentiation of which is believed to be vital in the pathogenesis of osteoporosis [39, 40]. NFATc1, c-Fos, and cathepsin K are the important regulators of osteoclast differentiation [40], with NFATc1 serving as one master transcription factor modulating osteoclast differentiation [41, 42]. Cathepsin K is a cysteine proteinase expressed predominantly by osteoclasts. Cathepsin K cleaves key bone matrix proteins and is believed to play an important role in degrading the organic phase of bone during bone resorption [43, 44]. c-Fos is another example of the transcription factor participating in the formation of osteoclasts [45, 46]. The gene and protein levels of them were upregulated by RANKL stimulation in RAW 264.7 cells, whereas CPT pretreatment notably reduced bone loss by decreasing the expression of cathepsin K, NFATc1, and c-Fos. Therefore, our study identified that CPT could improve osteoclasts by modulating the expression of osteoclast-specific genes and proteins.

## 5. Conclusions

Taken together, our data revealed that CPT could improve a series of diseases induced by menopause such as osteoporosis. CPT exerted an antiosteoclastogenesis effect by suppressing osteoclast-specific protein expression and protected against kidney failure.

## Data Availability

The data used to support the findings of this study are available from the corresponding author upon request.

## Abbreviation

SPF: Specific pathogen free
BMC: Bone mineral content
BGP: Bone gla protein
BMD: Bone mineral density
DMEM: Dulbecco's modified Eagle's medium
OVX: Ovariectomized
PMOP: Postmenopause osteoporosis
RANKL: Receptor activation of the nuclear factor kappa B ligand.

## References

[1] Yasser E. M. (2010). *Osteoporosis and Metabolic Bone Disease [127–142*.

[2] Vondracek S. F., Chen J. T., Csako G. (2004). Osteoporosis: pathophysiology and new drug development. *Clinical Reviews in Bone and Mineral Metabolism*.

[3] Cheng H. (2018). Bone and plasma citrate is reduced in osteoporosis. *Bone*.

[4] Meena M. K. (2021). *Bisphosphonates as Treatment and Prevention of Risk of Fractures in Postmenopausal Osteoporosis*.

[5] Kjeldsen S. E., Julius S., Hedner T., Hansson L. (2001). Stroke is more common than myocardial infarction in hypertension: analysis based on 11 major randomized intervention trials. *Blood Pressure*.

[6] Everitt A. V., Hilmer S. N., Brand-Miller J. C. (2006). Dietary approaches that delay age-related diseases. *Clinical Interventions in Aging*.

[7] Lobo R. A. (2014). Prevention of diseases after menopause. *Climacteric*.

[8] Carriero F. P., Christmas C. (2011). In the clinic. Hip fracture. *Annals of Internal Medicine*.

[9] Lewiecki E. M., Michael E. (2009). Current and emerging pharmacologic therapies for the management of postmenopausal osteoporosis. *Journal of Women’s Health*.

[10] Mahakala A., Thoutreddy S., Kleerekoper M. (2003). Prevention and treatment of postmenopausal osteoporosis. *Treatments in Endocrinology*.

[11] Mushtaq M. H. (2014). *A Review on Prevalence of Osteoporosis in Developing Countries*.

[12] Beckmann M. W., Jap D., Djahansouzi S. (2001). Hormone replacement therapy after treatment of breast cancer: effects on postmenopausal symptoms, bone mineral density and recurrence rates. *Oncology*.

[13] Hao Y., Zhao W., Zhang L. (2020). Bio-multifunctional alginate/chitosan/Fucoidan sponges with enhanced angiogenesis and hair follicle regeneration for promoting full-thickness wound healing. *Materials & Design*.

[14] Zhao W. (2020). *Dihydrotanshinone I Attenuates Plaque Vulnerability in ApoE-/- Mice: Role of RIP3*.

[15] Wang H., Pang W., Xu X., You B., Zhang c., Li D. (2021). Cryptotanshinone attenuates ischemia/reperfusion-induced apoptosis in myocardium by upregulating MAPK3. *Journal of Cardiovascular Pharmacology*.

[16] Chen L., Wang H.-J., Xie W., Yao Y., Zhang Y.-S., Wang H. (2014). Cryptotanshinone inhibits lung tumorigenesis and induces apoptosis in cancer cells in vitro and in vivo. *Molecular Medicine Reports*.

[17] Xia W. J., Chen Z. G., Zhang L. R. (2002). Experimental study on effect of cryptotanshinone in inducing differentiation of human bone marrow mesenchymal stem cells to neuron-like cells. *Chinese Journal of Integrated Traditional and Western Medicine*.

[18] You Y. (2015). *Effects of Cryptotanshinone on the Function of Bone Marrow Macrophages*.

[19] Zeng X. (2019). Preventive effects of a natural anti-inflammatory agent Salvianolic acid A on acute kidney injury in mice. *Food and Chemical Toxicology*.

[20] Lu J., Zhang Y., Liang J., Diao J., Liu P., Zhao H. (2021). Role of exosomal MicroRNAs and their crosstalk with oxidative stress in the pathogenesis of osteoporosis. *Oxidative Medicine and Cellular Longevity*.

[21] Guiming Y. (2020). *Association of Breastfeeding and Postmenopausal Osteoporosis in Chinese Women: A Community-Based Retrospective Study*.

[22] Cooper C., Campion G., Melton L. J. (1992). Hip fractures in the elderly: a world-wide projection. *Osteoporosis International*.

[23] Gullberg B., Johnell O., Kanis J. A. (1997). World-wide projections for hip fracture. *Osteoporosis International*.

[24] Zheng H., Feng H., Zhang W., Han Y., Zhao W. (2020). Targeting autophagy by natural product Ursolic acid for prevention and treatment of osteoporosis. *Toxicology and Applied Pharmacology*.

[25] Amend S. R., Uluckan O., Hurchla M. (2015). Thrombospondin-1 regulates bone homeostasis through effects on bone matrix integrity and nitric oxide signaling in osteoclasts. *Journal of Bone and Mineral Research*.

[26] Chambers T. J. (1998). The direct and indirect effects of estrogen on bone formation. *Molecular and Cellular Biology of Bone*.

[27] Zhao W., Yuan Y., Zhao H., Han Y., Chen X. (2019). Aqueous extract of Salvia miltiorrhiza Bunge-Radix Puerariae herb pair ameliorates diabetic vascular injury by inhibiting oxidative stress in streptozotocin-induced diabetic rats. *Food and Chemical Toxicology*.

[28] Zhao W. (2019). Structural characterization and in vitro–in vivo evaluation of effect of a polysaccharide from Sanguisorba officinalis on acute kidney injury. *Food & Function*.

[29] Shin J. H., Kim K.S. (2016). *Novel Use of Cryptotanshinone*.

[30] Feng Z., Zheng W., Li X. (2017). Cryptotanshinone protects against IL-1*β*-induced inflammation in human osteoarthritis chondrocytes and ameliorates the progression of osteoarthritis in mice. *International Immunopharmacology*.

[31] Shin J. H., Kim K. S., Lee I. K. (2016). *Use of Cryptotanshinone*.

[32] Wang W., Huang M., Hui Y., Yuan Y., Guo P., Wang X. (2019). Cryptotanshinone inhibits RANKL-induced osteoclastogenesis by regulating ERK and NF-kappaB signaling pathways. *Journal of Cellular Biochemistry*.

[33] He Y. Q. (2018). *Monotropein Attenuates Ovariectomy and LPS-Induced Bone Loss in Mice and Decreases Inflammatory Impairment on Osteoblast through Blocking Activation of NF-Κb Pathway*.

[34] Jochems C., Islander U., Erlandsson M., Verdrengh M., Ohlsson C., Carlsten H. (2005). Osteoporosis in experimental postmenopausal polyarthritis: the relative contributions of estrogen deficiency and inflammation. *Arthritis Research and Therapy*.

[35] Zhao W., Feng H., Sun W., Liu K., Lu J.-J., Chen X. (2017). Tert-butyl hydroperoxide (t-BHP) induced apoptosis and necroptosis in endothelial cells: roles of NOX4 and mitochondrion. *Redox Biology*.

[36] Zhao W., Feng H, Guo S, Han Y, Chen X (2017). Danshenol A inhibits TNF-*α*-induced expression of intercellular adhesion molecule-1 (ICAM-1) mediated by NOX4 in endothelial cells. *Scientific Reports*.

[37] Eyre D. R., Lee M. Y., Osborne W.

[38] Schepetkin I. (2010). Osteoclastic bone resorption: normal and pathological. *Annals of the New York Academy of Sciences*.

[39] Maeda T., Suzuki R., Ochi T.

[40] Yamakawa T., Okamatsu N., Ishikawa K. (2020). Novel gene Merlot inhibits differentiation and promotes apoptosis of osteoclasts. *Bone*.

[41] Ha K. J., Nacksung K. (2014). Regulation of NFATc1 in osteoclast differentiation. *Journal of Bone Metabolism*.

[42] Ballard A. (2020). The tethering function of mitofusin2 controls osteoclast differentiation by modulating the Ca2+-NFATC1 axis. *Journal of Biological Chemistry*.

[43] Lee J. H. (2011). Effect of water extract of rubi fructus in RANKL-induced osteoclast differentiation. *Journal of Physiology & Pathology in Korean Medicine*.

[44] Sakamoto H., Sakai E., Fumimoto R. (2012). Deltamethrin inhibits osteoclast differentiation via regulation of heme oxygenase-1 and NFATc1. *Toxicology in Vitro*.

[45] Hao H. (2006). Induction of c-Fos and NFATc1 during RANKL-stimulated osteoclast differentiation is mediated by the p38 signaling pathway. *Biochemical and Biophysical Research Communications*.

[46] Nosaka K. (2017). IL-17A inhibits osteoclast differentiation of RANKL-stimulated RAW264.7 cells by suppressing JNK phosphorylation and c-Fos expression. *Shika Igaku*.

